# Comparing the Effects of Two Tillage Operations on Beneficial Epigeal Arthropod Communities and Their Associated Ecosystem Services in Sugar Beets

**DOI:** 10.1093/jee/toy285

**Published:** 2018-09-27

**Authors:** Rudolph J Pretorius, Gary L Hein, Erin E Blankenship, Foster F Purrington, Robert G Wilson, Jeffrey D Bradshaw

**Affiliations:** 1 Department of Agriculture, Central University of Technology, Private Bag, Bloemfontein, Free State Province, South Africa; 2 Doctor of Plant Health Program, University of Nebraska–Lincoln, Lincoln, NE, USA; 3 Department of Statistics, University of Nebraska–Lincoln, Lincoln, NE, USA; 4 Department of Evolution and Ecology, The Ohio State University, 300 Aronoff Laboratory, Columbus, OH, USA; 5 Department of Agronomy and Horticulture, Panhandle Research and Extension Center, University of Nebraska–Lincoln, Scottsbluff, NE, USA; 6 Department of Entomology, Panhandle Research and Extension Center, University of Nebraska–Lincoln, Scottsbluff, NE, USA

**Keywords:** ground beetle, tillage, weed, predation

## Abstract

Beneficial arthropods provide important ecosystem services in terms of arthropod pest and weed management, but these services can be adversely affected by farming practices such as tillage. This study investigated the impact of two tillage operations (zone tillage and moldboard plow) on the activity density of several beneficial, epigeal arthropod taxa, and postdispersal weed seed and prey removal in sugar beet agroecosystems. In addition, four omnivorous ground beetle species were selected for a weed-seed choice feeding assay, whereas a single species was selected for a weed-seed age preference assay. Ground beetles were the most commonly collected taxon (via pitfall sampling), with only a few dominant species. Tillage operation did not affect ground beetle activity density; however, spider, centipede, and rove beetle activity densities were higher in the reduced-tillage treatment. Live prey consumption was similar between tillage practices, with more prey consumed during nocturnal hours. More weed seeds were consumed in the reduced-tillage treatment, whereas weed-seed preference differed between the four weed species tested [*Setaria pumila* (Poir.) Roem. & Schult., *Echinochloa crus-galli* (L.), *Kochia scoparia* (L.), and *Chenopodium album* (L.)]. In the weed-seed choice feeding assay, significantly more broad-leaf weed seeds (*C. album* and *K. scoparia*) were consumed compared with grassy weed seeds (*E. crus-galli* and *S. pumila*). No preference for seed age was detected for *E. crus-galli*, but *Harpalus pensylvanicus* (De Geer) preferred old *C. album* seeds over fresh seeds. Zone tillage is compatible with ecosystem services, providing critical habitat within agricultural ecosystems needed to conserve beneficial, edaphic arthropods.

Much research has focused on the role of beneficial arthropods (i.e., predators, parasitoids, and herbivores) in agroecosystems. There is consensus that these organisms should be conserved and enhanced to benefit from the ecosystem services they provide (Pimentel et al. 1992, Way and Heong 1994, Pickett and Bugg 1998, Altieri 1999, Cromar et al. 1999, Kendall 2003, Hooper et al. 2005, Zehnder et al. 2006, Fiedler et al. 2008, Holloway et al. 2008, Isaacs et al. 2008, Godfray et al. 2010, Benayas and Bullock 2012, Woodcock et al. 2014). The term ‘ecosystem services’ was defined by Daily (1997) as ‘the conditions and processes through which natural ecosystems, and the species that make them up, sustain and fulfill human life’. There is a multitude of ecosystem services, but arthropod-mediated ecosystem services (AMES) include the biological control of arthropod pests (predation and parasitism) and weeds (Isaacs et al. 2008), as well as pollination of several crops. In this way, AMES contribute to decreased pesticide input and increased crop sustainability and yield (Kendall 2003, Griffiths et al. 2008, Isaacs et al. 2008, Woodcock et al. 2014). Highlighting the importance of AMES, Losey and Vaughn (2006) estimated its value at almost $8 billion annually in the United States; $4.5 billion of which is due to the biological control of insect pests.

Several beneficial soil-dwelling arthropod taxa have been recorded from arable land, including spiders (Araneae), centipedes (Chilopoda), beetles (Coleoptera), harvestmen (Opiliones), earwigs (Dermaptera), true bugs (Heteroptera), neuropterans (Neuroptera), flies (Diptera), and ants (Hymenoptera). Many of these are polyphagous and have the capability to feed on a range of pest species (Kendall 2003). Farming operations, such as tillage, can have a profound impact on beneficial arthropod community structure and abundance (Stinner and House 1990, Weiss et al. 1990, Cárcamo et al. 1995, Heimbach and Garbe 1996, Clark et al. 1997, Altieri 1999, Andersen 1999, Holland and Luff 2000, Holland and Reynolds 2003, Kendall 2003) and, by implication, their associated ecosystem services. As such, the impact of farming practices on these organisms should be taken into account. Tillage can affect beneficial arthropod survival either through direct mortality or by modifying prey availability or the physical environment (Holland and Luff 2000, Kendall 2003, Holland 2004, Thorbek and Bilde 2004).

With the introduction of glyphosate-tolerant sugar beet varieties in 2008, the use of ‘zone tillage’ in sugar beet has increased. With zone tillage, residue from the previous crop is moved aside and a narrow zone (ca. 15–25 cm) is cultivated, where the new crop rows will be planted (Smith 2013). This form of reduced/conservation tillage is less intensive compared with more conventional tillage systems in the region that involve moldboard plowing where soil inversion takes place along with nearly complete burial of residue (Dickey et al. 1992). In addition, zone tillage lowers operational and labor costs (Smith et al. 1995) and protects against wind and water erosion. Zone tillage is practiced widely for sugar beet production in Nebraska, Colorado, and southern Wyoming, with 60% of the Nebraska sugar beet crop produced by this method (Smith 2013). This high adoption rate was primarily due to the ease of controlling weeds with a single active ingredient (glyphosate), reducing the need for additional cultivation practices (Smith 2013) or herbicide tank mixtures.

Unfortunately, glyphosate-resistant weed populations recently have been documented in many sugar beet production regions of the United States (Sandell et al. 2012, Heap 2014), necessitating an integrated approach to weed management in which beneficial arthropods could play an important role. Furthermore, an increased emphasis on improving sustainability of production systems places more emphasis on natural pest control as opposed to high-input agriculture that relies heavily on agrochemicals (Holland and Luff 2000).

Postdispersal weed-seed feeding (i.e., feeding on seeds shed from the parent plant) by vertebrates and invertebrates is widely recognized as an important contributing factor for weed management in agroecosystems (House and Brust 1989, Reader 1991, Cardina et al. 1996, Cromar et al. 1999, Kromp 1999, Menalled et al. 2000, Tooley and Brust 2002, Harrison et al. 2003, Honek et al. 2003, Westerman et al. 2003, Honek et al. 2005, Heggenstaller et al. 2006, Chauhan et al. 2010). By consuming weed seeds, the number of seeds surviving and germinating in the seed bank is reduced (Brust and House 1988, Crawley 1992, 2000, Cromar et al. 1999, Tooley and Brust 2002, Honek et al. 2003, Gallandt et al. 2005, Landis et al. 2005, Bohan et al. 2011). This can change weed community composition (Crawley 2000, Tooley and Brust 2002). Not only do weeds compete with the crop for nutrients, moisture, and sunlight, but they also can act as a secondary host to certain insect pest species (Hein and Johnson 2001, Capinera 2005).

Apart from affecting beneficial arthropod species assemblages and abundance directly, the ecosystem services, such as weed-seed consumption and invertebrate predation, also can be strongly influenced by tillage. For example, Brust and House (1988) reported that the rate of weed-seed removal by invertebrates (i.e., ground beetles, crickets, and ants) and rodents was twice as high in no-till soybeans compared with moldboard plowed fields. Cromar et al. (1999) also measured higher weed-seed consumption in no-till and moldboard plowed fields (averaging 32% weed-seed consumption) as opposed to chisel-plowed fields (averaging 24% weed-seed consumption). The observed differences in the degree of ecosystem services rendered between cultivation regimes might be a direct consequence of differing beneficial arthropod abundance brought about by the direct and indirect effects of tillage on their populations. It is also likely that the effects of tillage will have an impact on other ecosystem functions, such as arthropod predation, rendered by beneficial arthropods. However, the impact of tillage on other types of AMES, particularly on predation rates of prey, has received less attention.

It is imperative to assess the impact of farming operations, such as tillage, on ecosystem services and the organisms providing them, for the goal of identifying and developing better techniques to conserve and enhance these services (Altieri 1999). This is especially important considering the concerns over the long-term sustainability of our ecologically simplified agroecosystems (Altieri 1999). Several studies have investigated differences between species richness, abundance, and distribution of beneficial arthropods in various agroecosystems; however, few have investigated the degree of ecosystem function across management regimes (Griffiths et al. 2008).

We hypothesize that reduced tillage will improve the ecosystem function of resident beneficial arthropods in sugar beet agroecosystems in western Nebraska. Residue cover left on the soil surface in zone tillage systems should favor beneficial arthropods, leading to increased prey and postdispersal weed-seed removal rates as a result of their higher abundance. Our target taxon for inquiry was the Carabidae, as they are reportedly sensitive to cultivation (Holland 2004) and commonly found in the sugar beet agroecosystem (Pretorius et al. 2016). Therefore, carabids serve as bioindicators for monitoring the impact of cultivation on beneficial arthropods (Kromp 1999, Holland and Luff 2000). Furthermore, these insects are efficient generalist predators and weed-seed consumers.

## Materials and Methods

### Study Site

The study was conducted during the 2012 and 2013 field seasons at the University of Nebraska–Lincoln’s Mitchell Research Farm, located in the North Platte River Valley in western Nebraska (41° 56′ N; 103° 42′ W). The experimental plots were laid out in sugar beet plots established as part of a multiyear study investigating the long-term impact of crop rotations that include corn, dry beans, and sugar beet. This multiyear study was initiated in 2007 and included zone tillage, conventional tillage, and no-tillage conditions. However, for this particular study, only the zone tillage and conventional tillage plots were used. The plots for this experiment were established in a sugar beet field (cv. ‘Beta21RR25’) following corn cultivation. Each tillage practice was replicated five times in a randomized complete block design, with individual plots measuring 12 rows (6.7 m) by 65.2 m.

Prior to tillage operations, corn stalks were chopped by lightly disking the fields. Following stalk chopping, a zone tillage implement was used to establish the planting rows in the zone tillage plots. This implement consists of a single large coulter (to cut surface corn residue), positioned in front of each vertical shank (30.5 cm depth). Positioned directly behind each shank was a pair of wavy coulters that function to close the shank marks. Behind the wavy coulters are rolling baskets that compress and firm the seedbed to ensure good seed–soil contact. In contrast, the conventional tillage operation consisted of the use of a moldboard plow (ca. depth of 30.5 cm) after stalk chopping followed by two passes with a roller harrow to firm the seedbed for planting. Therefore, nearly all of the corn residue was buried below soil level in the conventional tilled plots. All research plots were treated twice with glyphosate early in the season for weed control.

### Beneficial Arthropod Activity Density

Beneficial epigeal arthropods were sampled throughout the field season (May–September) by means of pitfall trapping in the conventional tillage and zone tillage sugar beet plots (*n* = 6 traps per plot). During both years, the percentage of surface residue was estimated at two separate locations within each plot for both tillage systems by using the line–transect method (Shelton and Jasa 2009). Surface residue was measured on 4 August 2012 and 18 July 2013. To increase the capture efficiency, pairs of pitfall traps (for a total of three pairs per plot) were linked by means of a metal flashing barrier buried into the soil and running perpendicular to the sugar beet rows. The barrier measured circa 165 cm × 30 cm, with ca. 15 cm buried below soil level. They were installed in such a way that the edges of the barrier nearly touched the perimeter of the pitfall traps. The three pairs of pitfall traps within each plot were spaced 21.5 m apart from each other. Pitfall traps were constructed by making a hole in the soil with a 107-mm-diameter golf hole cutter and inserting a section of PVC pipe (76 mm diameter and 150 mm high) into each hole to prevent soil from collapsing. A small disposable plastic cup (147 ml capacity), containing a mixture (approximately 38 ml) of ethylene glycol and water (1:3 ratio) as a killing and preservation agent, was placed into each hole at the time of trap activation. A small amount (ca. 10 ml) of dishwashing liquid was also added to the master mixture (ca. 3.78 liters) to reduce surface tension. A tight-fitting plastic funnel (75 mm at the top and 25 mm at the bottom) was placed inside the PVC pipe and on top of each cup to ensure capture of soil arthropods. Each pitfall trap was subsequently covered with a plastic lid (250 mm diameter), leaving ca. 10 cm between the lid and soil surface for arthropods to enter. The plastic lids were secured to a 406.5 mm × 89 mm piece of wood with 127 mm bolts attached to each end that were used to secure the lid above the soil surface. Pitfall traps were left in the field for the duration of the growing season and capped with a tight-fitting lid when not activated.

The pitfall traps were activated six times each year (samples removed on 24 May, 8 June, 5 and 29 July, 14 August, and 11 September 2012 and on 30 May, 19 June, 10 and 30 July, 21 August, and 12 September 2013). The traps were left open for 5 d during each activation period. All samples were collected and stored in a cooler at 4–5°C until they could be processed. Although the emphasis was on ground beetles, several other taxa of beneficial epigeal arthropods were also sampled, including two additional beetle families (Staphylinidae and Coccinellidae), spiders (Order: Araneae), harvestmen (Order: Opiliones), and centipedes (Class: Chilopoda). These taxa were chosen based on their importance in agroecosystems as natural enemies of arthropod pests and weeds.

The seasonal abundance of four beneficial arthropod taxa (Carabidae, Chilopoda, Araneae, and Staphylinidae) were compared over the season (*n* = 6 sample dates) and between the two tillage practices by using a two-way ANOVA with repeated measures implemented in SAS PROC GLIMMIX, version 9.2, SAS (PROC GLIMMIX, SAS Institute 2008). The data from each pair of traps were combined (*n* = 3 traps per plot) due to low captures during certain sampling periods. For Chilopoda, the first sampling dates in both years were excluded from the analyses because few individuals were sampled during these periods. The data for the different taxa were fitted to one of two distributions (negative binomial or Poisson distribution) depending on the goodness of fit. Various covariance structures were considered for the data, and the appropriate structure (for each taxon within a particular year) was chosen based on the lowest values obtained for the Akaike information criterion containing a correction factor for finite populations, as discussed by Burnham and Anderson (2004). Significant differences between the means for both sample date and tillage effects were separated with a protected ad hoc least significant difference (LSD) test (α = 0.05). Marginal significant differences (*P* ≤ 0.08) are also discussed.

### Ground Beetle Species Richness and Diversity

Because of their higher activity density, ground beetles were identified to species level to compare species richness and diversity between treatments. Apart from measuring activity density, three diversity indices were calculated: species richness (*S*), the reciprocal of Simpson’s diversity index (1/*D*), and Simpson’s evenness (*E*). Simpson’s diversity index quantifies the diversity within a sample/habitat and is calculated as follows:

$$D=\sum p_{i}^{2}$$

where *p*_*i*_ is the proportion (from the total count of all species) of individuals for the *i*th species (Magurran 2004). Simpson’s diversity index accounts for both species richness (i.e., number of species in a sample) and evenness (i.e., the relative abundance of each species in the sample/habitat). The reciprocal of Simpson’s diversity index was used to calculate the diversity of ground beetles found for each tillage practice (1/*D*). The reciprocal index ranges on a scale from one to a maximum equal to the total number of species collected within the sample/habitat. The higher the value of this index is, the more even and diverse the species assemblage of the sample/habitat (Magurran 2004).

Simpson’s evenness was calculated as follows:

$$\begin{array}{l} E_{\text{1}/D}=(\text{1}/D)/S \end{array}$$

where *S* represents the number of species in the sample/habitat. Simpson’s evenness ranges on a scale from zero to one, with one indicating complete evenness (i.e., the proportion of each species is equal).

These diversity indices were calculated for each individual pitfall trap and for each sampling date separately. The data were compared between treatments by a two-way ANOVA with repeated measures, similar to that described for comparing activity density above. The data were fitted to either a negative binomial or Poisson distribution, depending on the goodness of fit. Significantly different means for sampling time and tillage effects were separated with an ad hoc LSD pairwise comparison test (α = 0.05).

### Postdispersal Weed-Seed Removal From the Field

Weed-seed removal rates by beneficial arthropods in sugar beet were compared by means of a split-plot experiment. The main plot treatments were tillage (conventional and zone tillage) and the split plot treatments were four different weed species. Weed species used included two grasses, yellow foxtail [*Setaria pumila* (Poir.) Roem. & Schult.] (Poales: Poaceae) and barnyardgrass [*Echinochloa crus-galli* (L.)] (Poales: Poaceae), and two broad-leaf weeds, kochia [*Kochia scoparia* (L.)] (Caryophyllales: Chenopodiaceae) and common lambsquarters (*Chenopodium album* L.) (Caryophyllales: Amaranthaceae). These species are common weeds in sugar beet production systems in the Central High Plains, and they have the capacity to reduce sugar beet yields (May and Wilson 2006). Furthermore, both lambsquarters and kochia act as secondary hosts for the sugar beet root aphid, *Pemphigus betae* Doane (Hemiptera: Aphididae), an important economic pest of sugar beet (Blackman and Eastop 2006, Hein et al. 2009). In addition, herbicide resistance has been observed in barnyardgrass (Carey et al. 1997, Talbert and Burgos 2007, Juliano et al. 2010), kochia (Guttieri et al. 1995, Foes et al. 1999, Cranston et al. 2001, Crespo et al. 2014), and common lambsquarters (Darmency and Gasquez 1990, Parks et al. 1996, Westhoven et al. 2008).

Seeds from each weed species were fixed to the bottom of modified petri dishes (100 × 15 mm) using double-sided sticky tape (Scotch 3M removable double-sided tape). Each petri dish contained 20 seeds of a single weed species for the two grasses and 30 seeds for the two broad-leaf weeds. Following attachment of the weed seeds to the sticky tape, fine gravel was added to coat the remaining sticky surface to prevent arthropods from becoming trapped. The seed dishes were placed into specially constructed exclusion cages, designed to keep out potential vertebrate seed feeders. These cages measured ca. 61 cm × 15 cm × 13 cm (length × width × height) and were constructed of galvanized wire screen (11 × 11 mm screen size). The sides of the cages were buried to ca. 4 cm below soil level. Four seed dishes, each containing the seeds of a different weed species, were randomly arranged into each exclusion cage. The seed dishes were buried into the soil, so the outside rims were flush with the soil surface to allow easy access. Three exclusion cages were randomly placed between the two center rows of each plot. To control for other factors that could contribute to seed removal (i.e., environmental factors such as rain, wind, etc.), a control cage was included in each plot. Control cages were of the same construction as experimental cages; however, they were completely covered with a fine, Lumite mesh screen material, preventing access to all potential seed feeders from outside the cages. This experiment was repeated on three separate dates during both the 2012 (2–13 July, 6–15 August, and 6–15 September) and 2013 (2–12 July, 14–23 August, and 6–16 September) growing seasons. The seed dishes were left in the field for approximately 10 consecutive days, and then the number of damaged and missing seeds was enumerated.

Because of low seed consumption rates during the first and last sampling dates of both years, data for each exclusion cage were pooled across the three sampling dates to arrive at the total number of seeds removed per cage for each weed species in that year. The proportion of seeds consumed for the 2 yr were fitted to a beta distribution and analyzed separately by means of a two-way ANOVA using the PROC GLIMMIX procedure in SAS version 9.2 (PROC GLIMMIX, SAS Institute 2008). This procedure was used to test for significant tillage and weed species differences and any interactions. Significantly different means among the two factors were separated using a protected LSD ad hoc test at the α = 0.05 level of significance.

### Weed-Seed Choice Feeding Assays

During the 2013 growing season, four ground beetle (Coleoptera: Carabidae) species, *Harpalus pensylvanicus* (De Geer), *H. erraticus* Say, *H. amputatus amputatus* Say, and *Amara carinata* (LeConte), were chosen for weed-seed feeding assays to determine weed-seed preference and consumption. These species were selected based on their ecological dominance during the 2012 field season. The beetles were hand collected from sugar beet research fields at the Mitchell research farm during their respective peak abundances. For this reason, all species were not tested at the same time. Prior to starting the experiment, all test insects were offered dehulled millet seed (*Panicum miliaceum* L.) (Poales: Poaceae) to ensure that they would accept food. Thereafter, they were provided with moisture but starved for 24 h before the experiment was initiated.

Feeding assays were conducted under choice conditions only, where ground beetles were presented a choice between the seeds of the same weed species used in the field experiment outlined earlier (yellow foxtail, barnyardgrass, kochia, and lambsquarters). Individuals were enclosed in plastic petri dishes (100 × 15 mm) containing a damp cotton wick as a source of moisture. A single beetle was introduced into each enclosure and presented with a total of 50 seeds from each weed species. Seed densities were selected based on a preliminary study where seed consumption was monitored over 48 h to ensure that the supply of seeds would not be exhausted during the experiment. The beetles were allowed 48 h to feed during the bioassay before they were collected and frozen at −20°C, dried at room temperature, and their mass recorded to the nearest 0.0001 g. The proportion of seeds destroyed, cracked, or visibly chewed upon for each weed species was recorded for each beetle. The assays were conducted in a growth chamber (27°C, 16:8 [L:D] h).

After the data were fitted to a Poisson distribution, the mean number of seeds consumed of each weed species by a given ground beetle species was compared by means of a one-way ANOVA. Significantly different means were separated using Tukey’s Honest Significant Difference multiple comparison ad hoc procedure at the α = 0.05 level of significance in SAS version 9.2 (PROC GLIMMIX, SAS Institute 2008). Preference for the seeds of either broad-leaf weeds (lambsquarters and kochia) or grass weeds (barnyardgrass and yellow foxtail) was also tested for the four ground beetle species by using orthogonal comparisons. To test whether beetle dry weight was correlated with the number of weed seeds consumed, a Pearson’s correlation was calculated (PROC CORR, SAS Institute 2008) for each of the four different ground beetle species.

### Field Predation Rates

To test for differences in the rate of pest removal between the two tillage systems, as well as any differences in pest removal during different times of the day, a field predation study was conducted concomitant to the weed-seed removal study. The experimental design was a split-plot in an RCBD with two tillage treatments (conventional and zone tillage) as the main plots and time of day (day and night) analyzed with a repeated measure analysis. Waxworm larvae (*Galleria mellonella* L.) (Lepidoptera: Pyralidae) were used as surrogate prey. Because the weed-seed removal experiment and the field predation experiment were conducted on approximately the same dates, the prey-removal study was also repeated three times throughout both 2012 (19–20 July, 15–16 August, and 14–15 September) and 2013 (18–19 July, 15–16 August, and 18–19 September). Following the protocol of Lundgren et al. (2006), waxworm larvae (of approximately equal size) were pinned onto triangular clay bases (Original Sculpey oven-bake clay) with #2 insect pins (Bioquip insect pins) to prevent their escape. The larvae were placed into the same vertebrate exclusion cages used in the postdispersal weed-seed removal experiment, with the clay bases buried below soil level. Three larvae were enclosed in each cage. Because predator activity can differ markedly between different times of the day due to the presence of nocturnal, diurnal, and crepuscular species, prey removal was monitored for 24 h from 07:00 a.m. to 06:00 a.m. The first observation period took place from 07:00 a.m. to 06:00 p.m. (day), whereas the second lasted from 07:00 p.m. to 06:00 a.m. (night). Larvae that were removed or killed were recorded as preyed upon. In addition, larvae with visible chewing scars, but which were still alive, were also scored as having been preyed upon. Any predators observed feeding on the larvae at the time of sampling were collected for further identification.

Predation of waxworm larvae was low during the first and last sampling dates of both years; therefore, the data from each exclusion cage were pooled across sampling dates for the total number of larvae removed per cage within each year (*n* = 9). Data for the 2 yr were analyzed separately by means of a two-way ANOVA by using the PROC GLIMMIX procedure in SAS version 9.2 (PROC GLIMMIX, SAS Institute 2008). The data were fitted to a binomial distribution that allowed for comparing the proportion of larvae removed between tillage practices and time of day. Significantly different means between tillage and time of day were separated using a protected LSD ad hoc test at the α = 0.05 level of significance.

### Weed-Seed Age Preference Assay

An experiment was conducted to determine whether one dominant ground beetle species showed preference for old (more than 5 yr old) versus fresh (collected from the parent plant in the current year of study) weed seeds. *H. pensylvanicus* was selected because it was the most active ground beetle species observed at the time of seed rain (mid-September to end September 2013). Individual beetles were hand collected from a sugar beet field and offered dehulled millet seed to ensure that they would accept food. Thereafter, the beetles were starved for a period of 24 h while being provided a moistened cotton wick. Beetles were then enclosed in plastic petri dishes (100 × 15 mm) containing a damp cotton wick for moisture. A single beetle was introduced into each enclosure and presented with a total of 50 seeds from each age group of either barnyardgrass or lambsquarters seeds (*n* = 24 beetles for each weed species). Enough seeds were included to prevent the beetles from consuming all the seeds of a particular age. The seeds from the two age groups were mixed before being placed into the feeding arenas. Barnyardgrass and lambsquarters were chosen because one was a grass and the other a broad-leaf weed, because of their high abundance in the area of research, because of difference in seed size between the two species, and because they seemed to be the most preferred weed species by *H. pensylvanicus* in the choice feeding assay described earlier. Visually, it was possible to distinguish between the seeds from the two age groups for both weed species, with fresh lambsquarter seeds containing a green seed coat, whereas the fresh barnyardgrass had a green tinge (older barnyardgrass had a yellowish brown color). The beetles were allowed 30 h to feed on the seeds before the experiment was terminated. This study was conducted under controlled circumstances in growth chambers (25°C, 12:12 [L:D] h).

The mean number of weed seeds consumed for the two different age groups within each weed species was compared with a one-way ANOVA after being fitted to a Poisson distribution. Significantly different means were separated by means of a protected LSD ad hoc procedure at the α = 0.05 level of significance in SAS version 9.2 (PROC GLIMMIX, SAS Institute 2008).

## Results

### Beneficial Arthropod Activity Density

During 2012, the average soil surface residue coverage was 8.2 and 81.2% in the conventional tillage and zone tillage plots, respectively. In 2013, 4.9 and 70.5% surface residue was measured in the conventional tillage and zone tillage plots, respectively. In total, 5,831 and 3,783 individual beneficial arthropods were sampled during the 2012 and 2013 growing seasons, respectively (Table 1). Due to the low abundance of harvestmen (*n* = 66 individuals for both years combined) and coccinellids (*n* = 28 individuals for both years), these taxa were not considered for any further analyses. Carabidae and Staphylinidae comprised 78% of the total beneficial arthropod abundance during the 2012 cropping season and 74% during the 2013 season.

**Table 1.** Total number of beneficial arthropods (by taxon) collected with pitfall trapping during 2012 and 2013

	Total number collected	
Beneficial arthropod taxon	2012^*a*^	2013^*a*^
Araneae (spiders)	702	703
Carabidae (ground beetles)	3,734	1,687
Chilopoda (centipedes)	506	249
Coccinellidae (lady beetles)	24	4
Opiliones (harvestmen)	23	43
Staphylinidae (rove beetles)	842	1,097
Total	5,831	3,783

^*a*^Total collected over six sampling dates within a particular year (*n* = 360 pitfall samples per year).

**Fig. 1.**
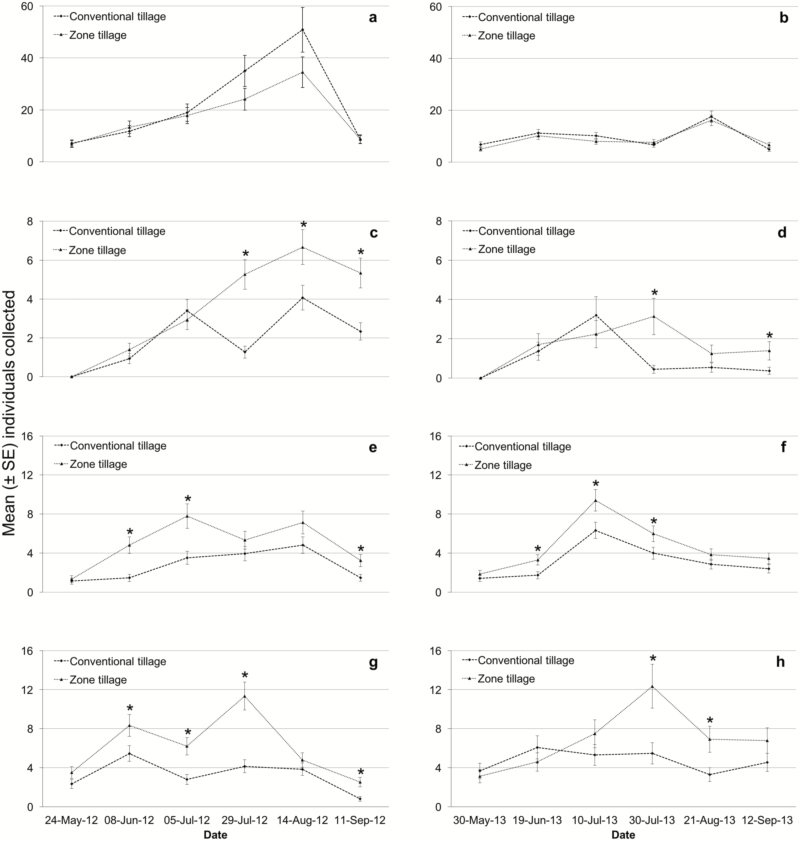
Mean (± SEM) number of ground beetles (a and b), centipedes (c and d), spiders (e and f), and rove beetles (g and h) collected during the 2012 (left-hand figures) and 2013 (right-hand figures) cropping seasons in sugar beets produced by means of two different cultivation practices (zone tillage and conventional tillage). Sample points on the x-axis indicate the date on which samples were collected from the field. Data analyzed by means of two-way ANOVA with repeated measures. An asterisk indicates significant difference between the two tillage practices within a date (α = 0.05).

The yearly mean numbers of ground beetles collected over the six sample dates in each tillage type are presented in Fig. 1a and b. A two-way ANOVA with repeated measures revealed that ground beetle activity density (for all species) was similar between the two tillage practices during both years (2012: *F*_1,4_ = 0.77, *P* = 0.43 and 2013: *F*_1,4_ = 0.51, *P* = 0.51). However, a significant effect of sampling time was observed during both seasons (2012: *F*_5,40_ = 52.01, *P* < 0.001 and 2013: *F*_5,40_ = 18.11, *P* < 0.001). The peak activity for both 2012 (mean ± SEM: 41.8 ± 5.6 beetles per trap) and 2013 (mean ± SEM: 16.8 ± 1.5 beetles per trap) seasons occurred on the fifth sampling date in August. The interaction between tillage practice and sampling time was nonsignificant during both years (2012: *F*_5,40_ = 1.27, *P* = 0.30 and 2013: *F*_5,40_ = 1.13, *P* = 0.36).

Both tillage practice (2012: *F*_1,4_ = 24.23, *P* < 0.008 and 2013: *F*_1,4_ = 10.4, *P* = 0.025) and sampling time (2012: *F*_4,40_ = 14.03, *P* < 0.001 and 2013: *F*_4,39_ = 5.01, *P* < 0.01) had a significant influence on centipede activity density during both years (Fig. 1c and d). Furthermore, a significant interaction between tillage practice and time was found each year (2012: *F*_4,40_ = 4.81, *P* = 0.03 and 2013: *F*_4,39_ = 3.69, *P* = 0.01). During both years, centipede activity densities were similar in both tillage systems up to the third sampling dates (5 July 2012 and 10 July 2013), but their activity was almost always significantly higher in the zone tillage treatment after this.

Each year spider activity density differed between the two tillage practices (2012: *F*_1,4_ = 31.03, *P* = 0.005 and 2013: *F*_1,4_ = 9.25, *P* = 0.04; Fig. 1e and f), with higher activity in the zone tillage plots (2012: 4.27 ± 0.33 vs 2.33 ± 0.21 spiders per trap and 2013: 4.07 ± 0.32 vs 2.73 ± 0.24 spiders per trap). Spider activity was also significantly affected by sampling time in both years with an increase and subsequent decrease in their abundance as the season progressed (2012: *F*_5,40_ = 14.97, *P* < 0.001 and 2013: *F*_5,39_ = 27.48, *P* < 0.001). However, their abundance peaked during the fifth sampling date in 2012 (mean ± SEM: 5.86 ± 0.72 spiders per trap) but earlier on the third sampling date in 2013 (mean ± SEM: 7.70 ± 0.68 spiders per trap). For this taxon, there was no tillage by sampling time interaction (2012: *F*_5,40_ = 1.64, *P* = 0.17 and 2013: *F*_5,39_ = 0.27, *P* = 0.93).

The mean number of rove beetles collected was marginally affected by both tillage (2012: *F*_1,4_ = 43.14, *P* = 0.003 and 2013: *F*_1,4_ = 3.81, *P* = 0.12) and sampling time (2012: *F*_5,40_ = 17.81, *P* < 0.001 and 2013: *F*_5,40_ = 4.99, *P* = 0.001; Fig. 1g and h). There were also marginally significant interactions between tillage and sampling time each year (2012: *F*_5,40_ = 2.29, *P* = 0.06 and 2013: *F*_5,40_ = 2.33, *P* = 0.06). With the exception of the first and fifth sampling dates (24 May and 14 August), rove beetle activity was significantly higher in the zone tillage plots during 2012. The marginal interaction was due to high beetle activity in the zone tillage treatment on July 29. During 2013, differences in rove beetle activity density between tillage systems were observed on the fourth and fifth sampling dates (30 July and 21 August) with higher activity for the zone tillage plots, but their numbers remained generally constant in the conventional tillage plots.

### Ground Beetle Species Richness and Diversity

With 5,421 total specimens (Table 1), ground beetles were the most commonly collected beneficial arthropods, comprising 41 species in 19 genera (Table 2). However, their numbers and species diversity were notably lower during 2013. Only a few ground beetle species dominated the samples. *H. erraticus*, *Elaphropus anceps* (LeConte), *H. pensylvanicus*, *A. carinata*, *Cicindela punctulata punctulata* Olivier, *Bembidion quadrimaculatum oppositum* Say, and *H. amputatus amputatus* comprised ca. 90% of the total diversity in 2012 (Table 2). These same species, with the exception of *H. amputatus amputatus* and the addition of *Bembidion tetracolum tetracolum* Say, *Clivina impressefrons* LeConte, and *Stenolophus comma* (Fabricius), comprised ca. 90% of the total ground beetle captures during 2013 (Table 2). Of these, *H. erraticus* and *E. anceps* made up 67% of this abundance in 2012 and 54% in 2013.

**Table 2.** Cumulative number of ground beetle species collected via pitfall trapping over six sampling dates during each field season of 2012 and 2013 in conventional tillage (CT) and zone tillage (ZT) plots

Species	2012^*a*^			2013^*b*^		
	CT	ZT	% Total	CT	ZT	% Total
*Agonum placidum* (Say)	6	5	0.29	7	12	1.13
*Amara carinata* (LeConte)	82	104	4.98^*c*^	24	45	4.09^*c*^
*Amara cupreolata* Putzeys	—	2	0.05	—	—	—
*Amara farcta* LeConte	6	23	0.78	—	1	0.06
*Amara quenseli quenseli* (Schönherr)	2	6	0.21	—	—	—
*Anisodactylus rusticus* (Say)	1	2	0.08	—	—	—
*Bembidion nitidum* (Kirby)	20	26	1.23	8	28	2.13
*Bembidion obscurellum obscurellum* (Motschulsky)	25	2	0.72	8	1	0.53
*Bembidion quadrimaculatum oppositum* Say	58	109	4.47^*c*^	61	71	7.82^*c*^
*Bembidion rapidum* (LeConte)	8	48	1.5	10	15	1.48
*Bembidion tetracolum tetracolum* Say	12	15	0.72	49	19	4.03^*c*^
*Bradycellus congener* (LeConte)	—	—	—	1	—	0.06
*Chlaenius tricolor tricolor* Dejean	—	2	0.05	2	—	0.12
*Cicindela cursitans* LeConte	—	1	0.03	—	—	—
*Cicindela punctulata punctulata* Olivier	101	69	4.55^*c*^	19	27	2.73^*c*^
*Cicindela purpurea audubonii* LeConte	1	—	0.03	—	—	—
*Clivina impressefrons* LeConte	3	2	0.13	27	33	3.56^*c*^
*Cratacanthus dubius* (Palisot de Beauvois)	—	—	—	—	1	0.06
*Dyschirius globulosus* (Say)	—	2	0.05	—	—	—
*Elaphropus anceps* (LeConte)	522	393	24.50^*c*^	316	213	31.36^*c*^
*Elaphrus clairvillei* Kirby	—	1	0.03	—	—	—
*Harpalus amputatus amputatus* Say	37	38	2.01^*c*^	23	16	2.31
*Harpalus caliginosus* (F.)	4	3	0.19	—	—	—
*Harpalus erraticus* Say	1,005	575	42.31^*c*^	209	175	22.76^*c*^
*Harpalus herbivagus* Say	11	41	1.39	2	10	0.71
*Harpalus opacipennis* (Haldeman)	1	—	0.03	—	—	—
*Harpalus pensylvanicus* (DeGeer)	103	162	7.10^*c*^	54	119	10.25^*c*^
*Harpalus reversus* Casey	5	12	0.46	3	1	0.24
*Harpalus somnulentus* Dejean	—	1	0.03	—	—	—
*Lebia bivittata* (F.)	1	5	0.16	—	—	—
*Lebia solea* Hentz	1	1	0.05	—	—	—
*Microlestes linearis* (LeConte)	2	3	0.13	1	5	0.36
*Pasimachus elongatus* LeConte	—	1	0.03	—	—	—
*Poecilus chalcites* (Say)	1	—	0.03	—	—	—
*Poecilus lucublandus* (Say)	1	7	0.21	1	3	0.24
*Poecilus scitulus* LeConte	4	1	0.13	3	2	0.3
*Pterostichus femoralis* (Kirby)	—	1	0.03	—	—	—
*Pterostichus melanarius melanarius* (Illiger)	1	—	0.03	2	2	0.24
*Pterostichus permundus* (Say)	1	1	0.05	—	2	0.12
*Stenolophus comma* (F.)	24	20	1.18	44	12	3.32^*c*^
*Stenolophus conjunctus* Say	1	—	0.03	—	—	—
Sum	2,050	1,684	100	874	813	100
Number of species	31	34	39	22	23	25

^*a*^A total of 3,734 ground beetles collected over six sampling dates.

^*b*^A total of 1,687 ground beetles collected over six sampling dates.

^*c*^Ground beetle species making up ca. 90% of the total captures within a specified year.

During 2012 and 2013, there was a significant effect of sample time on ground beetle species richness (Table 3). The effect of tillage on species richness was significant during 2012 (more species sampled in the zone tillage plots), but not during 2013. In both years, no interaction between sample date and tillage was observed (2012: *F*_5,40_ = 1.80, *P* = 0.14 and 2013: *F*_5,40_ = 0.61, *P* = 0.69). Sample time significantly affected the Simpson’s diversity in both years, but the tillage effect was only significant during 2012 (with higher diversity under the zone tillage treatment). No interaction between these effects was measured (2012: *F*_5,40_ = 1.72, *P* = 0.15 and 2013: *F*_5,40_ = 0.95, *P* = 0.46). Last, Simpson’s evenness was also significantly affected by sampling date during both years, but not by tillage, with no observed interaction (2012: *F*_5,40_ = 1.03, *P* = 0.41 and 2013: *F*_5,40_ = 1.37, *P* = 0.26; Table 3).

**Table 3.** Mean (± SEM) values for species richness (*S*), Simpson’s diversity (reciprocal: 1/*D*), and Simpson’s evenness (*E*) comparisons between the conventional tillage (CT) and zone tillage (ZT) systems during each of the six sampling dates for 2012 and 2013

	Species richness (*S*)		Simpson’s diversity (1/*D*)		Simpson’s evenness (*E*)	
	2012	2013	2012	2013	2012	2013
Tillage						
CT	2.82 ± 0.18	2.41 ± 0.12	1.93 ± 0.09	2.08 ± 0.07	0.72 ± 0.03	0.85 ± 0.01
ZT	3.44 ± 0.21	2.57 ± 0.12	2.56 ± 0.09	2.26 ± 0.07	0.77 ± 0.03	0.88 ± 0.01
df	1, 4	1, 4	1, 4	1, 4	1, 4	1, 4
*F*	10.7	0.93	30	3.25	1.11	4.87
*P*	0.03	0.39	0.005	0.15	0.35	0.09
Sample time						
1	2.19 ± 0.22	2.08 ± 0.19	2.05 ± 0.17	1.95 ± 0.12	0.90 ± 0.03	0.93 ± 0.02
2	3.26 ± 0.28	2.42 ± 0.20	2.65 ± 0.17	1.93 ± 0.12	0.83 ± 0.03	0.82 ± 0.02
3	3.14 ± 0.27	2.67 ± 0.21	2.18 ± 0.17	2.32 ± 0.12	0.74 ± 0.03	0.86 ± 0.02
4	4.06 ± 0.32	2.55 ± 0.21	2.61 ± 0.17	2.47 ± 0.13	0.65 ± 0.03	0.92 ± 0.02
5	3.89 ± 0.31	3.20 ± 0.23	1.81 ± 0.17	2.19 ± 0.12	0.49 ± 0.03	0.70 ± 0.02
6	2.58 ± 0.24	2.17 ± 0.19	2.14 ± 0.17	2.16 ± 0.13	0.87 ± 0.03	0.95 ± 0.02
df	5, 40	5, 40	5, 40	5, 40	5, 40	5, 40
*F*	9.68	3.82	3.56	2.74	50.30	21.32
*P*	<0.001	0.006	0.01	0.03	<0.001	<0.001

When comparing the most dominant species, it was evident that some species preferred the zone tillage system, whereas others were more abundant under the conventional tilled system (Table 4). However, most of the observed preferences were not consistent between years. One exception was that in both years, *H. pensylvanicus* showed higher activity in the zone tillage plots.

**Table 4.** Mean (± SEM) activity density of the most abundant ground beetle species collected by means of pitfall trapping in conventional tilled (CT) and zone tilled (ZT) sugar beets in western Nebraska

Ground beetle species	Treatment		df	*F*	*P*
	CT	ZT			
2012					
*Amara carinata* (LeConte)	2.62 ± 0.52	3.35 ± 0.64	1, 4	0.79	0.42
*Bembidion quadrimaculatum oppositum* Say	1.93 ± 0.27	3.63 ± 0.39	1, 4	12.75	0.02
*Cicindela punctulata punctulata* Olivier	2.93 ± 0.86	2.00 ± 0.60	1, 4	5.95	0.07
*Elaphropus anceps* (LeConte)	16.90 ± 2.61	12.46 ± 1.95	1, 4	3.59	0.13
*Harpalus amputatus amputatus* Say	1.23 ± 0.24	1.25 ± 0.25	1, 4	0.00	0.95
*Harpalus erraticus* Say	32.34 ± 6.82	16.69 ± 3.57	1, 4	4.85	0.09
*Harpalus pensylvanicus* (DeGeer)	3.43 ± 0.34	5.40 ± 0.42	1, 4	12.91	0.02
Other	4.68 ± 0.80	7.35 ± 1.20	1, 4	3.64	0.13
2013					
*Amara carinata* (LeConte)	0.79 ± 0.19	1.47 ± 0.29	1, 4	4.63	0.10
*Bembidion quadrimaculatum oppositum* Say	2.03 ± 0.26	2.37 ± 0.28	1, 4	0.76	0.43
*Bembidion tetracolum tetracolum* Say	1.54 ± 0.38	0.60 ± 0.19	1, 4	10.71	0.03
*Cicindela punctulata punctulata* Olivier	0.62 ± 0.17	0.88 ± 0.22	1, 4	1.12	0.35
*Clivina impressefrons* LeConte	0.90 ± 0.17	1.10 ± 0.19	1, 4	0.60	0.48
*Elaphropus anceps* (LeConte)	10.33 ± 1.27	6.96 ± 0.90	1, 4	19.80	0.01
*Harpalus erraticus* Say	6.83 ± 0.98	5.67 ± 0.84	1, 4	0.82	0.42
*Harpalus pensylvanicus* (DeGeer)	1.79 ± 0.28	3.96 ± 0.46	1, 4	16.80	0.01
*Stenolophus comma* (F.)	1.47 ± 0.22	0.40 ± 0.12	1, 4	15.92	0.02
Other	2.35 ± 0.36	3.73 ± 0.51	1, 4	5.02	0.09

### Postdispersal Weed-Seed Removal From the Field

The recovery rate from control cages during 2012 was as follows: 96.89% kochia, 99.67% yellow foxtail, and 100% for both barnyardgrass and lambsquarters in the conventional tillage plots. In the 2012 zone tillage plots, the recovery rates were as follows: 99% yellow foxtail, 99.33% barnyardgrass, 99.56% lambsquarters, and 100% kochia. The recovery rate from control cages during 2013 was as follows: 98.67% kochia, 99.33% yellow foxtail, 99.67% barnyardgrass, and 100% lambsquarters in the conventional tillage treatments. In the 2013 zone tillage plots, the recovery rates were as follows: 94.67% kochia, 98.44% lambsquarters, and 100% for both yellow foxtail and barnyardgrass. Due to these high recovery rates, there were no calculated corrections used for seed loss from sources within the exclusion cages.

**Fig. 2.**
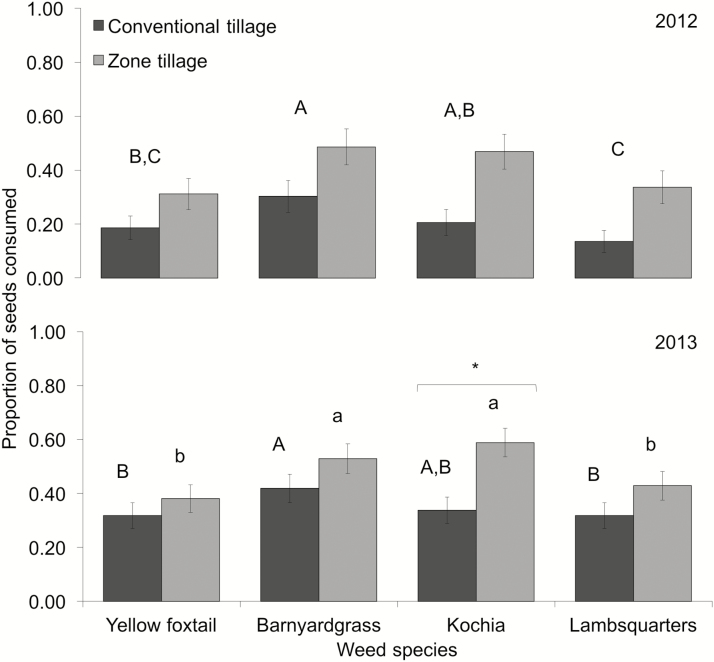
Mean proportion (± SEM) of weed seeds consumed during the 2012 (top figure) and 2013 (bottom figure) field seasons for four different weed species in conventional tillage and zone tillage plots by beneficial arthropods. For the 2012 field season, weed species with different letters differed significantly in the rate of their consumption by beneficial arthropods (LSD test, α = 0.05). For the 2013 field season, different letters indicate significant differences between weed species within a particular tillage system (capitalized letters = conventional tillage; lower case letters = zone tillage). Weed species with an asterisk indicates significant differences between tillage practices (LSD test, α = 0.05).

During 2012, the proportion of weed seeds consumed differed significantly between tillage practices (*F*_1,4_ = 10.62, *P* = 0.03) and weed species (*F*_3,24_ = 5.48, *P* = 0.005), with no observed interaction (*F*_3,24_ = 0.84, *P* = 0.49). A higher mean proportion of weed seeds were consumed in zone tillage treatments (mean ± SEM: 0.39 ± 0.05) compared with the conventional tillage treatments (mean ± SEM: 0.19 ± 0.03; Fig. 2). Furthermore, a significantly higher proportion of barnyardgrass was consumed compared with both yellow foxtail (*t =* 2.92, *P* = 0.008) and lambsquarters (*t =* 3.66, *P* = 0.001), whereas the consumption of kochia was higher compared with that of lambsquarters (*t =* 2.47, *P* = 0.02; Fig. 2).

During the 2013 field season, there was a significant tillage × weed species interaction, whereas the effect for tillage was marginally significant; weed-seed consumption in the conventional tilled plots approached the consumption levels observed in the zone tillage plots (tillage: *F*_1,4_ = 8.02, *P* = 0.05; weed species: *F*_3,24_ = 9.14, *P* < 0.001; tillage × weed species: *F*_3,24_ = 3.62, *P* = 0.03; Fig. 2). The interaction was due to the significantly higher consumption of kochia in the zone tillage treatment compared to the conventional tillage treatment (*t* = −4.16, *P* < 0.001; Fig. 2). Especially under the zone tillage treatment, the consumption rates of some of the weed species were notably high. Under this tillage treatment, 60% of the kochia seeds were consumed, whereas 54% of the barnyardgrass seeds were consumed.

### Weed-Seed Choice Feeding Assays

Weed species had a significant effect on the number of seeds consumed for all four beetle species (*A. carinata*: *F*_3,33_ = 25.55, *P* < 0.001; *H. amputatus amputatus*: *F*_3,33_ = 36.21, *P* < 0.001; *H. erraticus*: *F*_3,33_ = 47.41, *P* < 0.001; *H. pensylvanicus*: *F*_3,33_ = 91.45, *P* < 0.001; Table 5). Ground beetles preferred to consume lambsquarters seeds over other weed species (16 vs 5 or less: Table 5). Both *A. carinata* and *H. amputatus amputatus* preferred to consume broad leaves (kochia and lambsquarters) over grasses. In contrast, *H. erraticus* and *H. pensylvanicus* both had barnyardgrass seeds as the second most consumed weed species, albeit not statistically different from kochia in the case of *H. erraticus.* For all four beetle species tested, the mean number of broad-leaf weed seeds consumed (lambsquarters and kochia) was significantly higher (*P* < 0.05) compared with the grassy weeds (barnyardgrass and yellow foxtail), owing to the high consumption rate of lambsquarters (as determined by an orthogonal test). Averaged over all weed species, *H. erraticus* consumed an average of 4.94 seeds per individual per day, *H. pensylvanicus* 4.44 seeds per individual per day, *H. amputatus amputatus* 2.51 seeds per individual per day, and *A. carinata* 1.66 seeds per individual per day. Therefore, the overall number of weed species consumed by each of the four beetle species was not necessarily related to their size (dry weight). There was no correlation between beetle dry weight and the overall number of weed seeds consumed for *H. pensylvanicus* (*r* = −0.11, *n* = 12, *P* = 0.09), *H. amputatus amputatus* (*r* = 0.59, *n* = 12, *P* = 0.32), or *A. carinata* (*r* = 0.11, *n* = 12, *P* = 0.71). The only exception was with *H. erraticus* that displayed a negative correlation between beetle dry weight and the number of seeds consumed (*r* = −0.17, *n* = 12, *P* = 0.03).

**Table 5.** Mean (± SEM) number of seeds consumed for four weed species by four different omnivorous ground beetle species over a 48-h period

Weed	Ground beetle species				Overall mean
	*Amara carinata*	*Harpalus amputatus*	*Harpalus erraticus*	*Harpalus pensylvanicus*	
Barnyardgrass	0.32 ± 0.16a	0.22 ± 0.13a	10.26 ± 1.20a	8.12 ± 1.26a	4.91 ± 0.39a
Yellow foxtail	0.06 ± 0.07a	0.87 ± 0.29a	0.65 ± 0.24b	1.25 ± 0.35b	0.76 ± 0.13b
Kochia	3.12 ± 0.87b	6.26 ± 1.27b	8.06 ± 1.02a	2.11 ± 0.48b	5.36 ± 0.42a
Lambsquarters	6.82 ± 1.76c	10.20 ± 1.94c	19.63 ± 1.96c	21.79 ± 2.93c	15.78 ± 0.94c
Dry beetle dry weight (mg)	39.34 ± 2.21	23.02 ± 1.36	85.50 ± 5.10	56.27 ± 3.35	

Values within a column followed by different lower case letters indicate significant differences in the mean number of weed seeds consumed by a particular ground beetle species (Tukey’s Honest Significant Difference multiple comparison test, α = 0.05).

### Field Predation Rates

**Fig. 3.**
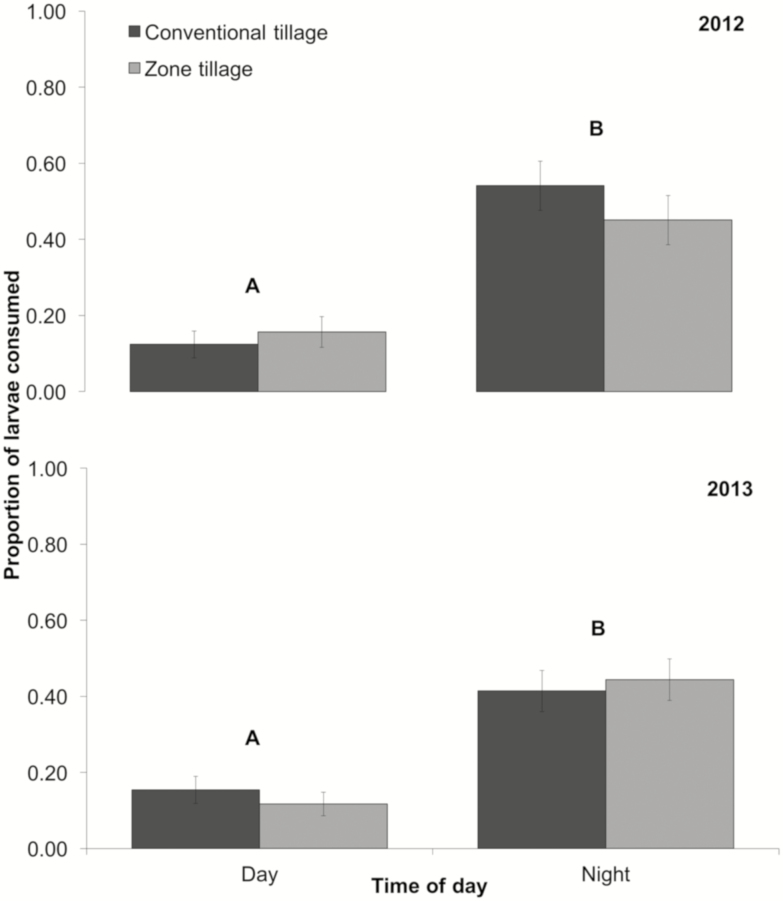
Mean proportion (± SEM) of waxworm larvae (*Galleria mellonella*) consumed during the 2012 (top) and 2013 (bottom) field seasons in the conventional tillage and zone tillage plots during different times of the day (day: 07:00 a.m.–18:00 p.m.; night: 19:00 p.m.–06:00 a.m.). Time periods with different letters are significantly different (LSD test, α = 0.05).

During 2012, the proportion of waxworm larvae removed from the exclusion cages differed significantly between the time of day, but not between tillage practices. There was no interaction between tillage or time of day (tillage: *F*_1,4_ = 0.01, *P* = 0.92; time of day: *F*_1,8_ = 36.56, *P* < 0.001; tillage × time of day: *F*_1,8_ = 1.28, *P* = 0.29). A higher proportion of larvae were consumed during the night compared with the day (Fig. 3). During the 2013 growing season, time of day had a significant impact on the proportion of larvae removed (tillage: *F*_1,4_ = 0.12, *P* = 0.74; time of day: *F*_1,8_ = 52.53, *P* < 0.001; tillage × time of day: *F*_1,8_ = 1.03, *P* = 0.34). As in the case of the 2012 growing season, the proportion of waxworm larvae consumed was highest at night (Fig. 3).

Despite the level of apparent predation in the field, few predatory arthropods were observed feeding on waxworm larvae. During 2012, only eight predatory arthropods were collected that fed on the larvae, including two individuals of the same species in the genus *Geocoris* (Hemiptera: Geocoridae), five ground beetles (Coleoptera: Carabidae), and one harvestman (Opiliones). The ground beetle species observed were *H. amputatus amputatus* (three individuals), *H. pensylvanicus* (one individual), and one unidentified species. During 2013, a total of eight predatory arthropods were also observed feeding on the waxworm larvae. However, in addition to these eight observations, seven waxworm larvae were observed being attacked by *Tetramorium caespitum* (L.) (Hymenoptera: Formicidae). Of the eight predators observed, one was a true bug, *Peritrechus convivus* (Stål) (Hemiptera: Rhyparochromidae), whereas ground beetles composed the remainder. The ground beetle observations included two individuals each for *E. anceps*, *B. quadrimaculatum oppositum*, and *H. erraticus* and one individual of *A. carinata*.

### Weed-Seed Age Preference Assay

*H. pensylvanicus* did not have a preference for seed age of barnyardgrass (*F*_1,23_ = 1.68, *P* = 0.21) with a mean (± SEM) consumption of 6.80 ± 0.78 fresh seeds and 7.79 ± 0.87 old seeds. However, this species did show a preference for old lambsquarters seeds (*F*_1,23_ = 104.34, *P* < 0.001), consuming a mean of 17.38 (SEM 1.91) fresh seeds versus 31.44 (SEM 3.31) old seeds.

## Discussion

The positive impact of epigeal natural enemies on pest insect populations in sugar beet agroecosystems has been reported previously (Hull and Gates 1953, Dunning et al. 1975, Landis and van der Werf 1997). Therefore, there are advantages in adopting farming practices that conserve and enhance beneficial arthropods in sugar beet (Kendall 2003). The interaction of tillage and arthropod conservation has received much attention (reviewed by Kendall 2003). Past studies comparing the effects of reduced-tillage methods on beneficial epigeal arthropods have provided contradicting results, which seems to be related to the group/species being compared. Research has shown increased abundance for certain taxa or species (e.g., House and Parmelee 1985, House 1989, House et al. 1989, Rice and Wilde 1991, Cárcamo 1995, Baguette and Hance 1997, Clark et al. 1997, Wilson-Rummenie et al. 1999, Langmaack et al. 2001, Holland and Reynolds 2003, Witmer et al. 2003, Sharley et al. 2008), whereas others observed decreased abundance (Rice and Wilde 1991, Baguette and Hance 1997, Clark et al. 1997, Holland and Reynolds 2003, Sharley et al. 2008) or no differences (Stinner and McCartney 1988, Rice and Wilde 1991, Cardina et al. 1996, Krooss and Schaefer 1998). However, in general, most have reported decreased abundance and activity density of beneficial epigeal arthropods in response to increased levels of cultivation.

In this study, centipede, spider, and rove beetle populations were mostly favored by the zone tillage system. Two plausible explanations for their higher activity densities under the zone tillage system could be attributed to the indirect effects of conventional tillage, such as decreased prey availability (and, conversely, more prey being available in the reduced-tillage system) or improved micro habitat (with more protection from intraguild predation) in the zone tillage system as a result of the higher percentage of crop residue. Decreased abundance of these taxa in response to plowing (and other forms of disturbance cultivation) has been reported elsewhere (Blumberg and Crossley 1983, Stinner and McCartney 1988, Krooss and Schaefer 1998, Cromar et al. 1999, Holland and Reynolds 2003, Sharley et al. 2008). For example, tillage has been shown to reduce populations of Collembola (Hendrix et al. 1986, Stinner and McCartney 1988, Miyazawa et al. 2002, Petersen 2002), a detritivorous group that constitutes an important component in the diets of generalist natural enemies (Blide et al. 2000, Petersen 2002). Higher organic matter on the soil surface resulting from crop residue in the zone tillage plots would probably support higher detritivore populations, thereby increasing predator abundance (House and Parmelee 1985). Reduced-tillage methods, when compared with inversion plowing, are also less likely to cause emigration of epigeal beneficial arthropods (Thorbek and Bilde 2004). The increased crop residue in zone tillage systems not only improves the habitable environment, but also alters the microclimatic conditions (e.g., soil humidity) to the possible benefit of these three taxa and their prey by preventing desiccation. Indeed, it is widely reported that increased organic residue on the soil surface enhances beneficial arthropod abundance in agroecosystems (Clark et al. 1993, Brust 1994, Miñarro and Dapena 2003, Thomson and Hoffmann 2007). Finally, direct mortality resulting from inversion plowing could also have contributed to decreased activity of centipedes, spiders, and rove beetles under this tillage system.

This study highlights the variability of generalist beneficial arthropod activity density for all taxa throughout the season. The implication of this temporally dispersed activity is that the combined activity of all taxa may ensure season-long pest regulation of various life stages of a range of insect pests (Kendall 2003). It assures that beneficial taxa will be present and have a regulatory impact on pest species during their immigration and establishment before specialists can respond numerically (Janssens and De Clerq 1990, Holopainen and Helenius 1992, Landis and van der Werf 1997). It is the diversity of species, coupled with their generalist feeding habits, that make generalist natural enemies important in pest management. This is despite the limited impact that any single beneficial species may have on pest populations or the varied abundance of individual beneficial species between years (Kendall 2003).

The impact of tillage regime on the activity density of ground beetles was negligible. This result may relate to two factors: ground beetle dispersal capabilities or the constraints associated with pitfall sampling. Ground beetles are excellent dispersers (Wallin and Ekbom 1988), and some species may more readily walk or fly between plots compared with other taxa. Pitfall trap captures measure the activity density of ground-dwelling invertebrates and not absolute density. Ground beetle abundance could be lower in the conventional tilled plots, but increased movement under this system could have enhanced the rate of capture (Shearin et al. 2007). Both scenarios could have led to the equal abundance observed between the two tillage regimes. However, it has been observed that some ground beetle species are favored by plowing, whereas others are affected negatively (e.g., House 1989, Cárcamo 1995, Clark et al. 1997, Holland and Reynolds 2003). Menalled et al. (2007) reported that the total activity density of ground beetles was higher in a conventional tilled system (moldboard plowing), but the activity density of weed-seed consumer species was higher in their no-till systems compared with the conventional tilled system. Shearin et al. (2007) also reported that moldboard plowing reduced granivorous ground beetle activity density significantly, whereas a predatory ground beetle species was negatively affected by all tillage systems investigated. In his review on the influence of tillage on epigeal predatory arthropods, Kendall (2003) concluded that because dominant ground beetle species often react differently to conventional and reduced-tillage systems, little or no differences in their activity density between these tillage types could be detected at the family level. Because of this, and as recommended by both Barney and Pass (1986) and Kendall (2003), abundant and functionally important taxa, such as ground beetles, need to be examined on the species level, rather than on the family level due to their differing feeding specializations and habitat preferences.

Focusing on the species level for ground beetles, it was clear that only a few species dominated numerically with seven dominant species during 2012 and nine during 2013, comprising ca. 90% of all captured individuals. More than half of the most abundant species sampled in this study have the capacity to consume both arthropod prey and weed seeds as shown with both field and growth chamber observations (Pretorius 2014), highlighting their importance to contributing to the sustainable management of these pests. Some of the most abundant ground beetle species showed a preference for the zone tillage system, whereas others preferred the conventional tillage system or remained unaffected. However, with the exception of *H. pensylvanicus*, their preference for a particular tillage system was not detectible between years. Both species richness and diversity of ground beetles were affected by tillage regime during 2012, as evidenced by differences between the cultivation practices during this season. This was, however, not the case during the 2013 field season, which can be ascribed to both a lower species diversity and a more even distribution of ground beetle species between the two tillage practices. Fewer ground beetles were also caught during 2013 compared with 2012, which can be ascribed to rainfall differences during the preceding years. Both the 2010 and 2011 seasons experienced high rainfall (giving rise to higher ground beetle numbers in 2012), whereas the 2012 and 2013 seasons were relatively dry (resulting in lower ground beetle numbers during the 2013 field season). Furthermore, the significant effect of sampling time on all three indices each year indicates that the species assemblage and activity density of the different species will also vary as the season progresses.

Increased crop residue left on the soil surface following cultivation has been shown to increase postdispersal weed-seed removal (Brust and House 1988, House and Brust 1989, Menalled et al. 2007). The results of this study agree with these findings as evidenced by higher postdispersal weed-seed removal rates in the zone tillage plots. These environments probably provide shelter for beneficial arthropods, and reduced soil disturbance also favor these organisms. Furthermore, there is evidence that the type of residue left on the soil surface can affect the number of weed seeds consumed. Postdispersal weed-seed removal is highest in plots with corn residue, compared with residue from other crops, such as wheat stubble (Cromar et al. 1999). In addition, differential selection between weed seeds under both field and laboratory conditions has been shown with this study, with similar findings reported by other authors (e.g., Jorgenson and Toft 1997, Tooley et al. 1999, Honek and Jarosik 2000, Honek and Martinkova 2001, Honek et al. 2003, 2005, Heggenstaller et al. 2006, Lundgren et al. 2006, Klimeš and Saska 2010, Meiss et al. 2010).

Under field conditions, barnyardgrass and kochia were favored most by seed-feeding arthropods (both seasons), demonstrating the capacity of these organisms to contribute to the destruction of both grass and broad-leaf weed seeds. Apart from kochia, a similar number of weed seeds were consumed between the two tillage practices for the remaining three weed species (2013) and might be a function of lower ground beetle activity density recorded during this season. Alternatively, reduced food choice, combined with greater mobility under plowed condition (House and All 1981, Crist et al. 1992), could also have contributed to this phenomenon. Cardina et al. (1996) also observed that the rate of postdispersal weed-seed removal can vary between seasons.

Ground beetles are renowned for their ability to consume weed seeds, which forms either a major component of their diets or a sporadic source of nourishment (Johnson and Cameron 1969). Due to the use of vertebrate exclusion cages, this study only investigated postdispersal weed-seed removal by arthropods. However, other organisms, such as rodents, could potentially consume a large proportion of weed seeds under field conditions (e.g., Brust and House 1988, Cardina et al. 1996, Harrison et al. 2003). No signs (e.g., droppings or the actual presence) of rodents were detected within the exclusion cages used in this study, suggesting that the cages worked to exclude vertebrates, and weed-seed removal was attributed to arthropod feeding. Furthermore, the pitfall samples showed few (or none) other important weed-seed feeders, such as crickets, isopods, ants, and slugs as recorded by Mittelbach and Gross (1984), Cardina et al. (1996), Hurst and Doberski (2003), O’Rouke et al. (2006), and Honek et al. (2009). None of these groups were observed feeding on the seeds in our seed choice tests in the field. However, several ground beetle species were regularly observed feeding within the exclusion cages on the weed seeds, and they constituted a majority of total pitfall samples; therefore, they probably constitute the key invertebrate seed feeders in sugar beets as noted for other systems (Brust and House 1988, Cromar et al. 1999, Honek et al. 2003, Westerman et al. 2003).

Seed choice in the laboratory assay differed from that observed in the field. Common lambsquarters were the most preferred weed species for all omnivorous ground beetle species under controlled conditions. Possible explanations for this difference could be that only four ground beetle species were tested in the growth chambers, whereas the omnivorous arthropod fauna under field conditions is composed of many species with a wide array of food items. Furthermore, Klimeš and Saska (2010) observed differences in weed-seed choice both within and between the larval and adult stages of ground beetle species and indicated that larval ground beetles might be more important weed-seed consumers than adults. This study did not determine the importance of ground beetle larvae in weed-seed consumption because larvae were rarely observed. Adis (1979) noted that pitfall traps would underestimate larval abundance as a result of larval size and movement speed.

In the laboratory choice feeding assay, the larger ground beetle species, *H. pensylvanicus* and *H. erraticus*, were more efficient consumers of the larger seeds of barnyardgrass compared to the two remaining smaller ground beetle species. Schoener (1971) reported that seed consumers would feed on the largest seeds they can handle. In contrast, the smaller size of lambsquarters did not prohibit their destruction by these relatively larger ground beetle species, and it has been shown by Lundgren and Rosentrater (2007) that some ground beetle species, such as *H. pensylvanicus*, actually preferred smaller, tougher seeds over larger seeds with weaker coats.

In contrast to the situation observed for weed-seed consumption, the number of surrogate prey (waxworm larvae) consumed did not differ between the tillage practices. This was despite the fact that pure predatory groups, such as spiders and centipedes, had a higher abundance in the zone tillage plots. Because the surrogate larvae used in this study were large (relative to size range for most of the predatory arthropods collected) and were often partially or totally consumed, it is probable that larger-sized predators were responsible for their consumption. Larger predatory arthropods, especially ground beetles which are known to be good dispersers (Wallin and Ekbom 1988), would find it easier to move between relatively smaller plots, possibly contributing to the lack of observable differences between tillage systems. As an example, the relatively large *H. erraticus* did not exhibit any differences in activity density between the tillage practices. Furthermore, the rate of predation on waxworm larvae in this study is similar to that observed in other studies (Lundgren et al. 2006). The fact that night-time predation rates were higher is to be expected, considering that many predatory arthropods are nocturnal (e.g., Vickerman and Sunderland 1975).

Considering the tremendous number of seeds that can be produced by certain weed species, it is likely that many seeds that enter the seed bank will not be immediately consumed. Therefore, age preference of seeds by arthropod consumers may be important. We found that *H. pensylvanicus* has no preference for seed age for barnyardgrass, whereas the reverse is true for lambsquarters. Two possible explanations why *H. pensylvanicus* chose old over fresh lambsquarters seeds might be related to seed toughness and strength and/or phytochemical seed protection. Lundgren and Rosentrater (2007) established that *H. pensylvanicus* preferred to feed on tougher and denser seeds (lambsquarters) under choice conditions, and seeds become denser with age as a result of dehydration. Fresh lambsquarters seeds used in this study still had their outer, softer seed coats attached, whereas these were not present on the older seeds. The seed coat might afford protection against granivory after they are shed from the parent plant, possibly due to phytochemical properties (Lundgren and Rosentrater 2007). A single ground beetle species (*H. pensylvanicus*) was provided with the seeds from only two weed species. It is possible that other ground beetle species have differing age preferences for weed seeds of various ages.

The advent of glyphosate-tolerant sugar beet varieties has facilitated the adoption of reduced-tillage practices, such as zone tillage. The results of this study also indicate that there is a strong incentive for adopting reduced-tillage practices in western Nebraskan sugar beet agroecosystems. Reduced tillage conserved many of the epigeal beneficial arthropod fauna examined in this study and increased the ability of these organisms to render crucial ecosystem services, such as weed-seed consumption. However, the advent of glyphosate resistance in weeds (e.g., kochia) currently casts doubt over the future of this reduced-tillage system. Importantly, zone tillage appears to be compatible with ecosystem services as measured in this study. Moreover, crop residue appears to provide the critical habitat within the agricultural ecosystem needed to conserve beneficial, edaphic arthropods. Therefore, future research concerning soil management should consider that some forms of tillage may be compatible with ecosystem services rendered by beneficial arthropods.
